# S6K1 Controls DNA Damage Signaling Modulated by the MRN Complex to Induce Radioresistance in Lung Cancer

**DOI:** 10.3390/ijms251910461

**Published:** 2024-09-28

**Authors:** Ali Calderon-Aparicio, Jun He, Nicole L. Simone

**Affiliations:** 1 Department of Radiation Oncology, Sidney Kimmel Comprehensive Cancer Center, Thomas Jefferson University, Philadelphia, PA 19107, USA; ali.calderonaparicio@jefferson.edu; 2 Department of Pathology, Sidney Kimmel Cancer Center, Thomas Jefferson University, Philadelphia, PA 19107, USA; jun.he@jefferson.edu

**Keywords:** radioresistance, S6K1, lung cancer, MRN complex, DNA repair

## Abstract

Radiation is a mainstay of lung cancer treatment; however, resistance frequently develops. Identifying novel therapeutic targets to increase radiation sensitivity is crucial. S6K1 is a serine/threonine kinase known to regulate protein translation which is associated with radioresistance, but the mechanisms involved are unknown. We proposed to determine whether S6K1 promotes radioresistance by regulating DNA repair in lung cancer. Colony formation, protein expression and proliferation were assessed. S6K1 was modulated pharmacologically by either PF-4708671 or genetically by Crispr-Cas9. Higher radioresistance levels in lung cancer cells were associated with lower phosphoactivation of MRN complex members, a key activator of radiation-induced DNA repair signaling. We also found lower levels of p-ATM, a target of the MRN complex, in more radioresistant cells, which was associated with a lower expression of γ-H2AX cafter radiation. Further, genetic and pharmacological S6K1 targeting sensitized lung cancer cells to low doses of radiation (*p* ≤ 0.01). Additionally, S6K1^−/−^ deletion increased the phosphoactivation of MRN complex members, indicating that S6K1 itself can shut down DNA damage regulated by MRN signaling. This is the first report showing that S6K1 inhibition radiosensitizes lung cancer cells by decreasing MRN complex-regulated DNA repair signaling. Future studies should evaluate the role of S6K1 as a target to overcome radioresistance.

## 1. Introduction

Lung cancer remains the deadliest type of cancer worldwide. In the USA, 611,720 cancer deaths will occur in 2024, with lung cancer being responsible for 21% of these deaths [1]. Although radiotherapy is one of the mainstay treatments for lung cancer, patients develop disease progression. Several biological factors contribute to this phenomenon, including cancer stem cells, genetic mutations, immune-surveillance deficiency and metabolic dysfunctions. Furthermore, clinical trials have evaluated radiation dose escalations in patients to achieve improved therapeutic outcomes; however, these randomized trials have demonstrated increased toxicity without improved efficacy [2,3,4]. Therefore, identifying new targets to improve radiation efficacy that promote greater therapeutic benefits after radiation is a priority for clinical therapy in lung cancer care.

More than half of cancer patients are treated with radiotherapy, which kills tumor cells by directly and indirectly by inducing DNA damage, including cytotoxic DNA double-strand breaks (DSBs) [5]. Cells respond to DNA damage by activating checkpoint signaling and DNA repair pathways, collectively termed the DNA damage response (DDR). When the damage is excessive, the DDR triggers programmed cell death. However, mutations or alterations in the expression of DDR proteins predispose to cancer and determine the response to genotoxic treatments such as chemo and radiotherapy, mainly allowing cancer cells to escape from death and develop resistance and higher malignancy [6].

Ribosomal protein S6 kinase 1 (S6K1) is a serine-threonine kinase involved in angiogenesis, cell cycle progression and metastasis. S6K1 activation involves phosphorylation at both residues T389 and T229 [7], which is associated with the development of resistance to chemo and radiotherapy [7,8,9,10,11,12]. Here, the S6K1 inhibition has shown a radiosensitizer effect in several types of tumors. For example, in prostate and oral cancer, S6K1 downregulation sensitizes tumor cells to radiation, causing arrest in the cell cycle and DNA damage [13,14]. Furthermore, in lung cancer, S6K1 knockdown enhanced its radiosensitivity by delaying radiation-induced DSBs repair, G2/M checkpoint arrest, and inducing apoptosis [15,16].

The MRE11-RAD50-NBS1 complex (MRN complex) is among the first sensors of DNA breaks and coordinates the DDR [17]. The MRN complex recognizes and binds DSBs, the main genomic lesion caused by radiotherapy, which induces activation of their key members. MRN activation involves phosphorylation of RAD50, which renders the complex active to phosphorylate the target ataxia telangiectasia mutated protein (ATM), an essential component of DDR. Then, ATM initiates various cellular responses, including cell cycle control, transcription, and DNA repair [17]. In addition to these protumor effects, the overexpression of MRN complex members is positively correlated with poor response of cancer patients to radiotherapy [18].

Previous reports suggest a role for S6K1 in the regulation of DDR. Here, S6K1 overactivation by genetic deletion of PTEN can suppress DNA repair after irradiation by downregulating the MRN complex member MRE11 in colon cancer cells. Moreover, these cells show greatly diminished proficiency for DNA repair via the error-free homologous recombination (HR) repair pathway [19]. MRE11 is highly unstable in PTEN-deficient cells, but it can be restored by S6K1 inhibition [19]. Furthermore, S6K1 regulates Mdm2-mediated p53 ubiquitination after genotoxic stress and presents a route for cells to incorporate the metabolic/energy cues into the DNA damage response [20]. Due to the importance of MRN complex signaling for repairing DNA damage caused after radiotherapy and the suggested inhibitory role of S6K1 on DNA damage repair induced by genotoxic treatments, we investigated whether targeting S6K1 controls the MRN complex-regulated response of lung cancer cells against DNA damage after radiotherapy. In addition, we determined the role of S6K1 in the radioresistance development in our cell models. Our results showed that S6K1 inhibition decreased the colony formation ability of lung cancer cells after radiation, an effect that was accompanied by an activation of MRN complex signaling and a higher level of DNA damage after radiation. Furthermore, the genetic deletion of S6K1 increased the phosphoactivation of members of the MRN complex and its main target p-ATM. Thus, the inhibitory effect of S6K1 on the MRN complex activity could lead to the accumulation of genetic abnormalities, increasing malignancy and resistance of tumors to DNA-damaging therapies like radiation.

## 2. Results

### 2.1. MRN Complex Signaling Is Diminished in Endogenously Radioresistant Cells

**Figure 1:**
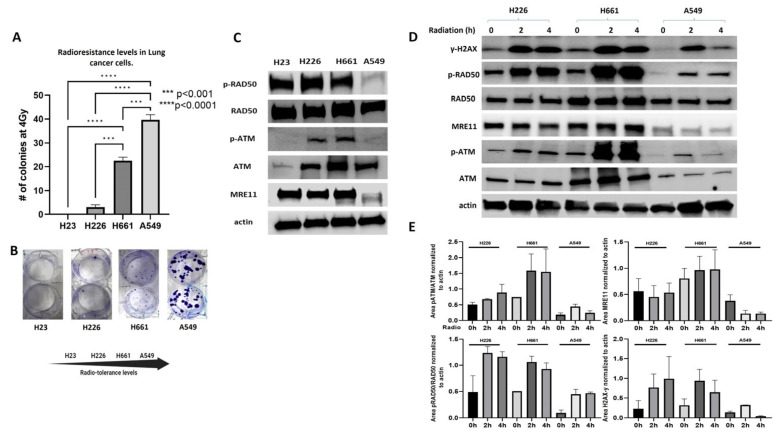
MRN complex signaling is diminished in endogenously radioresistant cells. (**A**) Lung cancer cells show different radiosensitivity after irradiating them with a dose of 4 Gy (*p* < 0.001). (**B**) Irradiated cells were stained with a crystal violet solution and visible colonies photographed. (**C**) Immunoblot assays showing the phosphorylation levels of MRN complex members in lung cancer cells. (**D**) Cells were irradiated with a dose of 10 Gy for 2 and 4 h and the expression changes were compared with non-radiated controls by immunoblot assays. The expressions of p-RAD50, MRE11 and p-ATM are decreased in more endogenously radioresistant cells compared to controls before and after radiation. (**E**) Quantitation of expression changes normalized to actin. Normalization was performed using ImageJ version 1.54h. *** denotes a *p* value < 0.001 and **** <0.0001.

We assessed the endogenous radioresistance levels of lung cancer cells by colony formation assays and compared them with the phosphorylation levels of RAD50 and ATM, key components of the DDR activation after radiation [7]. First, lung cancer cell lines were chosen to model radiation response by clonogenic assays and survival fraction calculation. In our experiments, the H23 cells were the most sensitive, followed by the cell lines H226 and H661, with the most resistant being the A549 cells (Figure 1A,B). Surprisingly, lower p-RAD50 levels were detected in the A549 cells, the most radioresistance cell line evaluated. Interestingly, the expression of MRE11, another member of the MRN complex, was also decreased in A549 cells (Figure 1C). Since our data showed an association between radioresistance and inhibition of MRN complex signaling, indicating that cells intrinsically more radioresistant show lower phosphoactivation of the MRN complex, we hypothesized that a decreased MRN pathway signaling could lead to accumulation of DNA damage and aberrant mutations in the genome, which would increase malignancy and resistance to radiotherapy in lung cancer.

To prove this hypothesis, we studied both the phosphoactivation of MRN complex members and the induction of DNA damage markers before and after radiation in lung cancer cells, to associate these levels with intrinsic radioresistance. The expression levels of p-RAD50 and MRE11, key components for MRN activation, were decreased in endogenously radioresistant cells A549, along with p-ATM, a downstream target of MRN, before and after radiation, compared with the more radiosensitive cells H226 and H661 (Figure 1D,E). Here, we found that even though the phospho-levels of MRN members were slightly higher in the H661 cells compared to the most sensitive cells (H226), this difference was not significant (Figure 1E). We only observed a great reduction in the A549 cells, indicating that this highly radioresistant cell harbors the lower activation of MRN signaling compared to the more sensitive cells, H226 and H661. In a similar way, the expression of γ-H2AX, a very well-known marker of DNA damage, was decreased after radiation in the A549 cells, the cell model with lower phosphoactivation of RAD50, ATM and lower expression of MRE11. Therefore, we conclude that the endogenous radioresistance in lung cancer cells is affected at least partially by the activation levels of MRN complex signaling, with cells harboring the lower levels of MRN signaling having less DNA damage, as measured by the expression of γ-H2AX and more radioresistance.

### 2.2. S6K1 Inhibition Sensitizes Lung Cancer Cells to Rradiation

**Figure 2:**
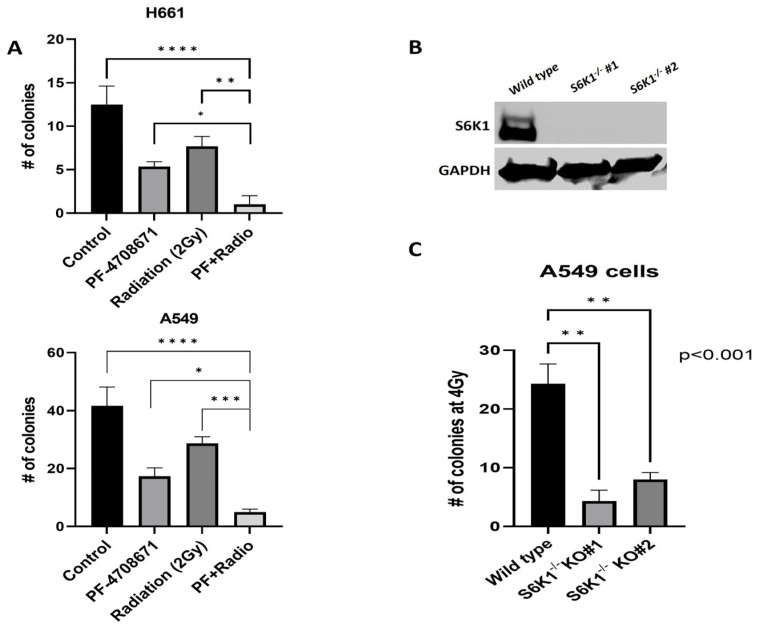
Genetic and pharmacological targeting of S6K1 decreases radioresistance in lung cancer cells. (**A**) Lung cancer cells were seeded in 6-well plates in triplicate and left attached overnight. Then, cells were exposed to PF-4708671 (5μM) or DMSO for 24 h before radiation. Cells were exposed to 2 Gy of radiation and were grown until visible colonies were formed (2 weeks). PF-4708671 was kept for the whole experiment. Colony formation was determined by clonogenic assays. (**B**) S6K1^−/−^ KO A549 cells were lysated, and the deletion of S6K1 gene was confirmed by Western blot. (**C**) Colony formation was determined by clonogenic assays in S6K1^−/−^ KO A549 cells after irradiation with a dose of 4 Gy. The S6K1 deletion dramatically sensitizes A549 cells to radiation. * denotes a *p* value < 0.01, ** and *** <0.001 and **** <0.0001.

As the activation of S6K1 could have a role in radiation resistance in lung cancer, we studied the phospho-levels of S6K1 in our panel of cell lines. Our results indicated that S6K1 activation, measured by the phosphorylation in T389, was increased in the most endogenously radioresistant cells, A549 (Figure S1). Moreover, there was an increase in the phosphorylation of the ribosomal protein S6 (S6), the main known target for S6K1 kinase, in both resistant cells H661 and A549 (Figure S1). We also checked the levels of p-S6K1 after radiation and found the more radioresistant cells, A549 and H661, harbor the highest levels of activation of this target, while the most sensitive H23 cells have the lowest, confirming the association between the levels of activated S6K1 and radioresistance (Figure S2). To further evaluate the role of S6K1 in radioresistance, we pre-treated A549 and H661 cells with DMSO (control vehicle) or PF-4708671, a highly specific inhibitor of S6K1 and performed colony forming assays before and after radiation. PF-4708671 is a piperazinyl-pyrimidine analogue which was the first specific S6K1 inhibitor to be reported. It was shown that PF-4708671 suppresses the phosphorylation of the protein S6, while having little or no effect on other S6K1-related kinases [21]. While a decrease in colony formation in cells treated with PF-4708671 or radiation alone was observed, a more significant reduction was noted in cells pre-treated with PF-4708671 plus radiation, indicating that S6K1 inhibition sensitizes lung cancer cells to radiation (Figure 2A). To confirm that the radiosensitizing effects of PF-4708671 were due to the specific inhibition of S6K1 and not to off-target effects of the PF-4708671 inhibitor, we selected the most endogenously radioresistant A549 cells and deleted the *S6K1* gene by Crispr-Cas9 (S6K1^−/−^ KO (knockout) cells) for further clonogenic assays. First, we confirmed the genetic deletion of S6K1 by Western blotting (Figure 2B). As expected, the colony formation was dramatically decreased in two different clones of S6K1^−/−^ KO cells compared to the *wild type* (*p* < 0.001) after radiation (Figure 2C). Although we observed a clear decrease in the colony number in non-radiated S6K1^−/−^ cells compared to the *wild type* controls (Figure S3), the reduction in colonies in S6K1^−/−^ cells plus radiation was far higher, showing that the S6K1 genomic deletion has, in fact, a radiosensitizing effect on lung cancer cells. Thus, this set of data confirms the pharmacological or genetic targeting of S6K1-sensitized lung cancer cells to radiotherapy.

### 2.3. S6K1 Modulates DNA Damage Signaling by Regulating Activation of MRN Complex Members

**Figure 3:**
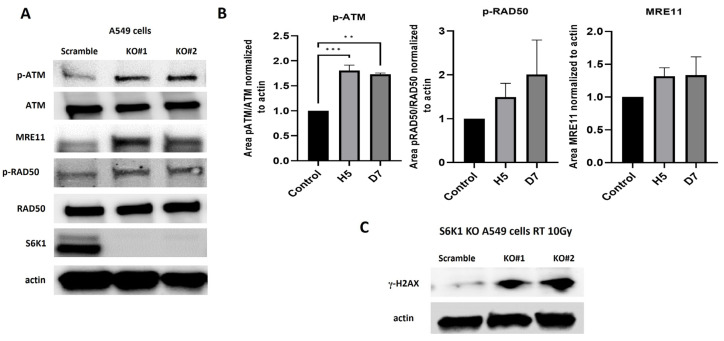
S6K1 impairs DNA damage signaling by inhibiting MRN complex activation. (**A**) Expression levels of MRN complex members were determined in S6K1^−/−^ KO and wild type cells by Immunoblot. The S6K1 genetic deletion increased the activation of p-RAD50, p-ATM and expression of MRE11, compared to wild type controls. (**B**) Quantitation of expression changes normalized to actin. (**C**) S6K1^−/−^ KO and wild type A549 cells were irradiated with a dose of 10 Gy. At 4 h after irradiation, cells were lysated and γ-H2AX expression was determined by Immunoblot. ** denotes a *p* value < 0.05 and *** <0.005.

Our data showed that MRN complex signaling activation is diminished in cells that are more inherently radioresistant and that S6K1 inhibition cooperates to overcome resistance to radiation. Therefore, we hypothesize that a nexus between S6K1 and MRN could exist to promote radioresistance. To confirm this association, we analyzed the phosphoactivation of members of the MRN complex signaling in S6K1^−/−^ A549 cells. Our results showed that S6K1^−/−^ cells exhibited upregulation of p-ATM, p-RAD50 and MRE11 compared to *wild type* controls (Figure 3A,B), confirming the inhibitory role of S6K1 on MRN complex signaling. Moreover, irradiation was shown to cause more DNA damage signaling in S6K1^−/−^ cells compared to controls, observed by a significant overexpression of the DNA damage marker γ-H2AX in KO cells after radiation (Figure 3C). Thus, our data confirmed that S6K1, at least partially, controls DNA damage by regulating MRN complex signaling.

### 2.4. S6K1 Overexpression Increases Resistance to Radiation and Impairs Activation of MRN Signaling

**Figure 4:**
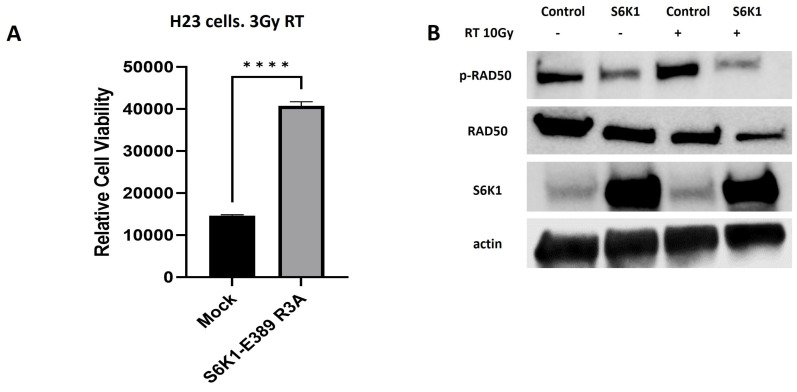
S6K1 overexpression increases radioresistance by impairing MRN complex signaling. (**A**) S6K1 overexpression increases radioresistance and proliferation in H23 cells. Cells were transfected with a plasmid expressing a constitutively active form of S6K1 (addgene #8991) or an empty vector. At 24 h after transfection, 5000 cells/well were seeded in 96-well plates and left attached overnight. Cells were irradiated with a dose of 3 Gy and incubated for 72 h. Proliferation was performed by Alamar Blue. (**B**) H23 cells overexpressing an empty vector or an active S6K1 were lysated and the expression levels of MRN members were investigated before radiation and at 4 h after. **** denotes a *p* value < 0.0001.

To confirm the regulatory role of S6K1 on the development of radioresistance, we overexpressed a constitutively active form of S6K1 in the radiosensitive cells H23 and performed proliferation assays after irradiating the cells. Our results showed that the overexpression of S6K1 significantly increases the radioresistance of H23 cells compared to controls (mock) (Figure 4A). In fact, this higher resistance was accompanied by a reduction in the phosphoactivation of RAD50 (Figure 4B, strengthening our previous data showing a role for S6K1 in the regulation of MRN complex activation. Thus, we confirmed that S6K1 promotes radioresistance by impairing MRN complex-regulated DNA damage signaling.

## 3. Discussion

Cancer cells possess an unstable genome leading to genetic alterations and tumor heterogeneity, allowing them to accumulate aberrant mutations that induce defects in the DDR machinery by impairing DNA repair mechanisms and conferring resistance to DNA-targeting antitumor treatments [22]. Aberrant activation of different cell-signaling pathways that promote genetic alterations in cancer has been reported [17,23,24]. Due to the modulatory role of S6K1 on the induction of resistance to chemotherapy observed in several cancers, we investigated whether this kinase modulates radioresistance in lung cancer. Our data showed that S6K1 inhibition by PF-4708671 sensitized lung cancer cells to radiation, compared to non-radiated controls or cells treated only with PF-4708671 (Figure 2A). PF-4708671 is a drug which inhibits S6K1 with high specificity, although the precise mechanism of action is unknown. A previous report characterized the effects of PF-4708671 and showed that it inhibits only S6K1 with great specificity, even at micromolar concentrations, without affecting other related kinases such as mTOR or AKT [21]. This study also showed that PF-4708671 induced a phosphorylated state of S6K1; however, this hyperphosphorylated S6K1 is inactive since it produced a dramatic reduction in the phosphorylation of S6, the main target of S6K1 [21]. It is possible that PF-4708671-induced phosphorylation is a result of inhibiting the negative-feedback loop in which S6K1 phosphorylates IRS-1 (insulin receptor substrate-1). S6K1 inhibits the PI3K/mTOR pathway by phosphorylating IRS-1 on multiple serine residues, which promotes its degradation, suppressing PI3K pathway activity [21,25]. In any case, PF-4708671 seems to render an inactive S6K1 protein in a phosphorylated state, and this inactivation renders lung cancer cells sensitive to radiation. To confirm our hypothesis and avoid any side effects exerted by the inhibitor, we genetically deleted the *S6K1* gene by Crispr-Cas9 technology. Our results showed, as before, that the S6K1 deletion sensitized lung cancer cells to radiation. Even though we saw a reduction in the colony number in non-radiated S6K1^−/−^ KO controls and cells undergoing radiation alone, it was clear that the *S6K1* deletion significatively reduced the colony number after radiation compared to each control. Therefore, we conclude that *S6K1* deletion sensitized lung cancer cells to radiation (Figure 2C and Figure S3).

Furthermore, we discovered that S6K1 controls DNA repair by inhibiting the activation and expression of the MRN complex members p-RAD50 and MRE11. In fact, the endogenous expression of these phosphoproteins was decreased in cells more radioresistant, before and after irradiation. Thus, impairing S6K1 signaling leads to a decrease in the activation of p-ATM (Figure 1D,E and Figure 3A), which is responsible for promoting repair mechanisms of DSB produced by radiotherapy [17]. Hence, S6K1 activation could inhibit DNA repair mechanisms by modulating MRN complex signaling, allowing cancer cells to escape death after radiation and accumulate genetic abnormalities, which makes them more resistant to therapy. This hypothesis was supported by the fact that we saw more DNA damage in S6K1 KO cells after radiation, as evaluated by the expression of DNA damage markers such as γ-H2AX (Figure 3C). Regarding the concern about why more DNA damage was seen in S6K1^−/−^ KO cells even though MRN signaling was higher, it is possible that this repair system is not able to fix all the damage and these KO cells go to die after a high dose of radiation (10 Gy), such as the used here. This observation was supported by the fact that the knockout of S6K1 or its pharmacological inhibition greatly decreased the cell colony number after radiation, showing the radiosensitizing role for S6K1 inhibition.

S6K1 is best known for its regulatory roles in protein synthesis and cell growth by phosphorylating its primary substrate, ribosomal protein S6, upon mitogen stimulation. Moreover, the roles of this kinase in various aspects of cancer progression, such as epithelial–mesenchymal transition, cancer stemness and drug resistance, have been shown [26]. Furthermore, S6K1 promotes inflammation and a decrease in lifespan, confirming the tumorigenic roles of this kinase [27,28]. Therefore, the therapeutic targeting of S6K1 alone or in combination with traditional chemotherapies or other microenvironmental-based drugs such as immunotherapy may represent promising approaches against cancers [26].

**Figure 5:**
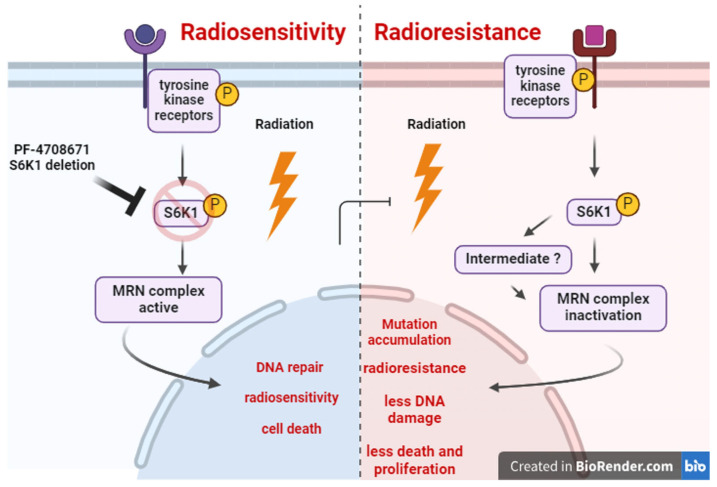
Working model showing the regulation of the AKT/mTOR/S6K1 pathway on the MRN complex signaling activation. After radiation, S6K1 phosphorylated and activated directly, or via an intermediate, inhibits MRN complex signaling, leading to defects in DNA repair, mutation accumulation, apoptosis inhibition and radioresistance. Furthermore, the inhibition of S6K1 by pharmacological inhibition or genetic deletion renders the MRN complex active, which promotes the DDR mechanisms, promoting DNA repair or apoptosis if the damage is extensive, making radiotherapy more effective. Diagram created using Biorender.

Supporting this idea is the fact that S6K1 is overexpressed and activated by tumor-promoting cell signaling pathways. For example, the PI3K/AKT/mTOR pathway is overactivated in cancer, and it is well known that this pathway activates S6K1. The overactivation of this pathway occurs in part by a downregulation of AKT inhibitors such as PTEN [29]. PTEN deficiency is observed often in cancer, which induces AKT activation. A previous study showed that this AKT activation has a suppressor role for DNA repair via S6K1, effects accompanied by a reduced expression of MRE11, RAD50, and NBS1 in colon cancer [19]. Thus, we hypothesize that PI3K/AKT/mTOR signaling could over activate S6K1, and then this kinase, either directly or by activating a downstream intermediate, could mediate DDR inhibition by impairing MRN signaling, allowing cells to accumulate aberrant mutations that increase radioresistance and malignancy (Figure 5).

## 4. Materials and Methods

### 4.1. Cell Culture

All cell lines used in this study came from the American Type Culture Collection (ATCC, Manassas, VA, USA). The cell lines H23, H661 and H226 were cultured in RPMI 1640 medium (Gibco; Waltham, MA, USA). The A549 cells were cultured in F12K (ATCC; Manassas, VA, USA). All mediums were supplemented with 10% serum and 1% penicillin–streptomycin solution. Cells were maintained at 37 °C and 5% CO^2^ in a humidified atmosphere and harvested with trypsin 0.25%.

### 4.2. Western Blot

Cells were lysed with RIPA buffer (Thermo Fisher #89901; Waltham, MA, USA) supplemented with a phosphatase–protease inhibitor cocktail (Thermo Fisher #1861281; Waltham, MA, USA) and kept on ice for 30 min. The lysates were cleared by centrifugation, and protein expression was assessed by Western blot, as reported previously [30]. The signal was detected in a ChemiDoc imaging system using chemo-luminescence reagents (Thermo Scientific #34075; Waltham, MA, USA).

### 4.3. Colony Formation Assay

Cell suspensions were seeded into six-well plates and left to attach overnight. After attachment, PF-4708671 (Medchemexpress LLC #50-187-3356, Monmouth Junction, NJ, USA) or DMSO was added 24 h before irradiation and kept until the end of the experiment. The cells were irradiated at the indicated doses and then incubated for 10 days or until colonies were visible. At this point, the cells were fixed with 100% methanol and stained with a 0.5% crystal violet solution (Sigma, St. Louis, MO, USA) for 20 min. The wells were washed with tap water and the stained colonies were counted and photographed.

### 4.4. Crispr-Cas9 Cells

S6K1 was genetically deleted in A549 cells through Crispr-Cas9 tools from SYNTHEGO (Redwood city, CA, USA). The gRNA sequence (AAUGAAAGCAUGGACCAUGG) used was targeted against exon 2. Two knock out clones were selected for further experiments. Cells transfected with a mock gRNA were used for controls (wild type).

### 4.5. Cell Transfection

The radiation-sensitive H23 cells were transfected with an empty vector (Addgene #10883, Watertown, MA, USA) or with a vector expressing a modified cDNA expressing a constitutively active form of S6K1 (pRK7-HA-S6K1-F5A-E389-R3A) (Addgene #8991, Watertown, MA, USA) [31,32]. Briefly, cells were seeded in 60 mm^3^ and left to attach overnight. When the cells reached a confluency between 60 and 80%, they were transfected by using the FuGENE^®^ 4K Transfection reagent from Promega (#E5912, Madison, WI, USA).

### 4.6. Proliferation Assays

The H23 cells transfected with S6K1 or mock treatments were incubated for 24 h and detached with trypsin. Then, 100 μL of medium containing 7500 cells was seeded into each well in 96-well plates. At 24 h after seeding, the cells were irradiated at the indicated doses and incubated for 72 h. At the end of the experiment, 10 μL of CellTiter-Blue^®^ Cell Viability Assay reagent (Promega #G8082, Madison, WI, USA) was added to each well and incubated at 37 °C for 2 h. The fluorescence was read in a microplate reader with excitation/emission wavelengths of 560 nm/590 nm. Values are represented as relative proliferation in relation to control.

### 4.7. Statistical Analysis

Values are shown as mean values ± S.E.M. All experiments were conducted in biological triplicate with representative data shown. Comparisons to determine statistical significance between groups were performed by Anova followed by a Bonferroni post hoc test using GraphPad Prism 9.5 software (San Diego, CA, USA). Differences between samples with a *p*-value of ≤0.1 were considered statistically significant.

## 5. Conclusions

Our data showed that S6K1 inhibition sensitized lung cancer cells to radiation by decreasing MRN complex-mediated DNA damage signaling. This inhibition allowed the accumulation of genetic mutations in cancer cells and the escape of cell-death mechanisms, which could increase malignity and resistance to radiotherapy. This study lays the basis for the design of future research projects oriented to evaluate the effects of S6K1 as a radiosensitizer in several types of cancer.

## Data Availability

The original contributions presented in the study are included in the article; further inquiries can be directed to the corresponding authors.

## Supplementary Materials

The following supporting information can be downloaded at: https://www.mdpi.com/article/10.3390/ijms251910461/s1.
