# Supplementary material for: S6K1 Controls DNA Damage Signaling Modulated by the MRN Complex to Induce Radioresistance in Lung Cancer
Source: Int J Mol Sci. 2024 Sep 28;25(19):10461. doi: 10.3390/ijms251910461 (PMC11477310; doi:10.3390/ijms251910461)
Supplement: Supplementary file 1 [file ijms-25-10461-s001.zip › ijms-3186904-supplementary.pdf]

Figure S1.

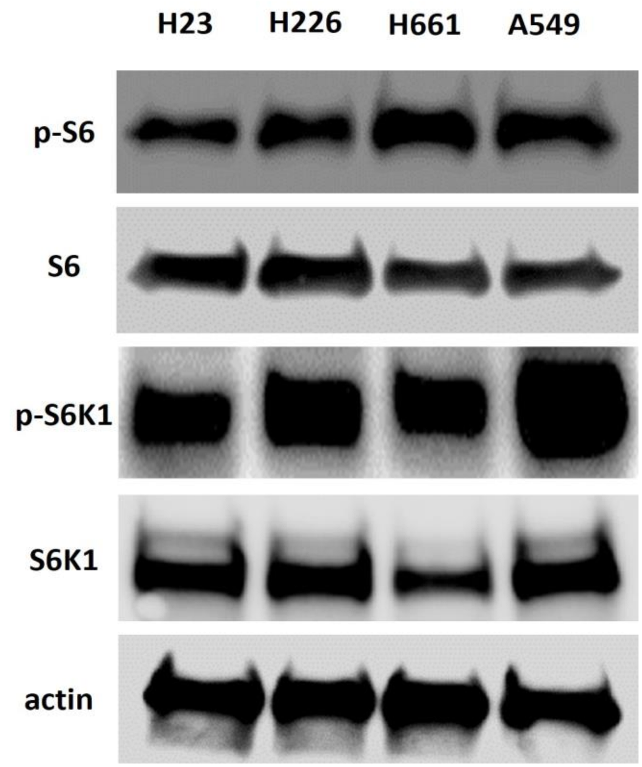

Figure S1. **Levels of p-S6 and p-S6K1 increase in most radioresistant cells**. Lung cancer cells show expression levels of p-S6K1 and p-S6 by immunoblot experiments.

Figure S2.

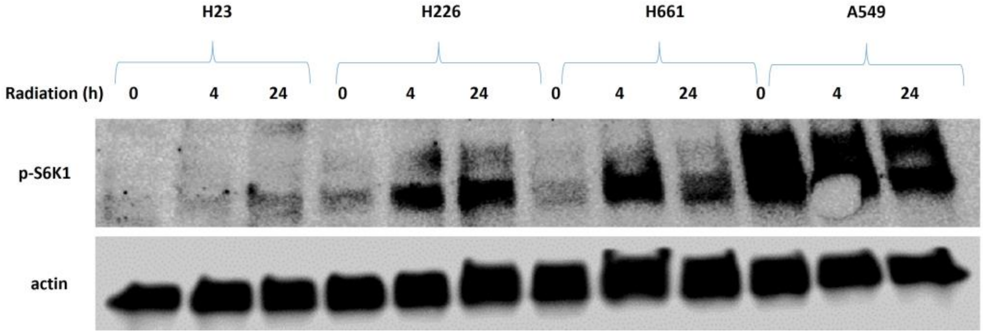

Figure S2. Levels of p-S6K1 increase after radiation. Cells were seeded in tissue plates and radiated with a single dose of 10 Gy. Then, cells were harvested at the indicated times and protein expression was evaluated by Immunoblot as described in methods section.

Figure S3.

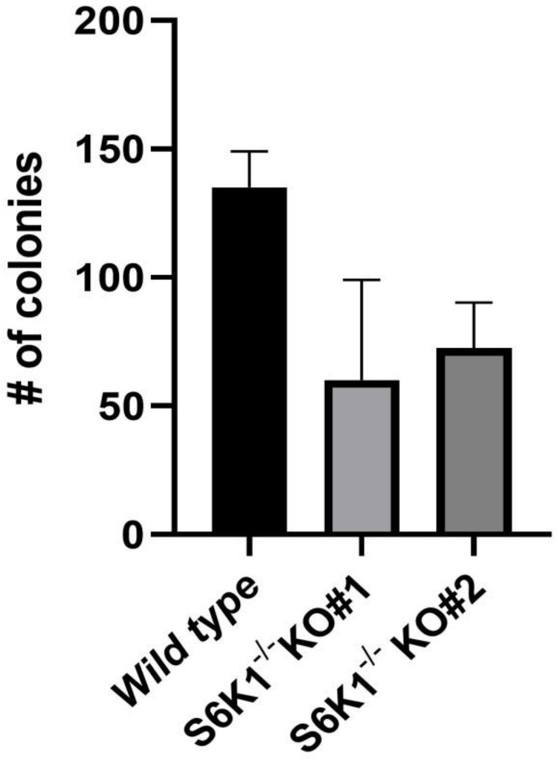

Figure S3. Genetic targeting of S6K1 decreases colony formation in lung cancer cells. Colony formation was determined by clonogenic assays in S6K1$^{-/-}$ KO A549 cells and compared with *wild type* controls.
